# Influenza Chimeric Protein (3M2e-3HA2-NP) Adjuvanted with PGA/Alum Confers Cross-Protection against Heterologous Influenza A Viruses

**DOI:** 10.4014/jmb.2011.11029

**Published:** 2020-12-02

**Authors:** Chaewon Kwak, Quyen Thi Nguyen, Jaemoo Kim, Tae-Hwan Kim, Haryoung Poo

**Affiliations:** 1 Infectious Disease Research Center, Korea Research Institute of Bioscience and Biotechnology (KRIBB), Daejeon 344, Republic of Korea; 2 Department of Biosystems and Bioengineering, KRIBB School of Biotechnology, University of Science and Technology (UST), Daejeon 34113, Republic of Korea

**Keywords:** Influenza virus, universal vaccine, adjuvant, cross-reactivity

## Abstract

Vaccination is the most effective way to prevent influenza virus infections. However, conventional vaccines based on hemagglutinin (HA) have to be annually updated because the HA of influenza viruses constantly mutates. In this study, we produced a 3M2e–3HA2–NP chimeric protein as a vaccine antigen candidate using an *Escherichia coli* expression system. The vaccination of chimeric protein (15 μg) conferred complete protection against A/Puerto Rico/8/1934 (H1N1; PR8) in mice. It strongly induced influenza virus-specific antibody responses, cytotoxic T lymphocyte activity, and antibody-dependent cellular cytotoxicity. To spare the dose and enhance the cross-reactivity of the chimeric, we used a complex of poly-γ-glutamic acid and alum (PGA/alum) as an adjuvant. PGA/alum-adjuvanted, low-dose chimeric protein (1 or 5 μg) exhibited higher cross-protective effects against influenza A viruses (PR8, CA04, and H3N2) compared with those of chimeric alone or alum-adjuvanted proteins in vaccinated mice. Moreover, the depletion of CD4^+^ T, CD8^+^ T, and NK cells reduced the survival rate and efficacy of the PGA/alum-adjuvanted chimeric protein. Collectively, the vaccination of PGA/alum-adjuvanted chimeric protein induced strong protection efficacy against homologous and heterologous influenza viruses in mice, which suggests that it may be a promising universal influenza vaccine candidate.

## Introduction

Influenza viruses are single-stranded RNA viruses that can be classified into subtypes based on the hemagglutinin (HA) and neuraminidase (NA) of their surface proteins [1]. They belong to the *Orthomyxoviridae* family and undergo highly variable antigenic mutations similar to those of other RNA viruses [2, 3]. Influenza viruses cause respiratory diseases in humans and cause between 290,000 and 650,000 deaths every year [4]. The main host that spreads influenza viruses is wild birds, which are primary reservoirs for most influenza A viruses [5]. At present, vaccination is the most effective method available for preventing influenza virus spread and infections [6, 7].

Current influenza virus vaccines are predicted and composed of influenza A (H1N1 and H3N2) and influenza B viruses. They are highly strain- and HA-specific, but do not induce cross-reactive antibodies [8, 9]. The drawback of forecasted influenza virus vaccines is weak immune responses against antigenic variants of influenza viruses [10] because the vaccines could be mismatched with circulating influenza viruses due to rapid antigenic shift and drift in these viruses [11, 12]. Thus, annual vaccination is necessary, but much effort is required to manufacture influenza virus vaccines. Moreover, influenza viruses have produced four pandemics within the past 100 years; the Spanish flu (1918), Asian flu (1957), Hong Kong flu (1968), and swine flu (2009), and the Spanish flu caused approximately 50 million deaths in 1918 [13]. Because of these outbreaks and risks, many researchers have tried to develop a universal influenza vaccine (UIV) to induce broad protections against diverse influenza viruses [14-18].

To date, the HA2 (stem region), extracellular domain of matrix protein 2 (M2e), and nucleoprotein (NP) are considered the major targets of UIV [15, 19, 20]. HA1 (head region) has a critical role in binding to receptors leading to entry into the host cells [21, 22]. On account of this, it has been used as an essential element to develop commercial influenza vaccines, but many studies reported the importance of HA2-specific antibodies providing heterosubtypic protection [23-25], and researchers focused on a conserved region of HA2 across influenza A subtypes [26]. In particular, a non-neutralizing antibody induced by HA2 could promote antibody-dependent cellular cytotoxicity (ADCC) related to cross-protection against influenza viruses [27]. However, immune responses generated against the HA2 are only effective to confer protection against a low dose of heterologous influenza virus infections and are insufficient against a high dose of heterosubtypic influenza viruses [28]. Thus, many efforts have been made to develop another UIV target candidate such as M2 including M2e, a well-conserved protein in influenza viruses [29]. The M2 protein as a vaccine antigen induces a high level of M2-specific antibodies and confers protection against both homologous and heterologous influenza viruses [30, 31]. Moreover, M2 contains B- and T-cell epitopes that promote humoral and cellular immune responses in vaccinated mice [32-36]. Nevertheless, M2e-specific antibodies showed the limitations of neutralization and prevention of influenza virus infections, and they are mainly responsible for virus clearance following infections [28, 37].

Accumulated reports demonstrated that cytotoxic T lymphocytes (CTL) are critical for cross-protection against influenza viruses [38, 39], and nucleoprotein (NP), a highly conserved region of the influenza virus, contains epitopes that induce CD8^+^ T cell responses that kill virus-infected cells [40, 41]. The vaccination of mice with NP antigen induces CD8^+^ T cell responses, leading to marked cross-protection against lethal challenges of heterologous influenza viruses [41-43]. However, none of these conserved antigens has been licensed as a UIV so far despite numerous trials. Thus, the improvement of vaccine formulation is necessary to enhance vaccine efficacy and cross-protection against divergent influenza viruses.

Recently, numerous efforts have been made to generate a combination of recombinant proteins (HA2 and M2e) and Toll-like receptor ligand (flagellin)-fused recombinant protein, and virus-like particles to develop a new universal influenza vaccine antigen. It has been reported that recombinant proteins (HA2 and M2e) and flagellin-fused protein targeting M2e and HA2 could induce the cross-reactivity against heterologous influenza viruses [44-46]. However, the heterosubtypic cross-reactivity of adjuvanted chimeric protein (3M2e-3HA2-NP) has never been investigated.

The use of vaccine adjuvants is also a promising strategy to enhance cross-protection against antigenically diverse influenza viruses [28]. A previous report demonstrated that the use of a complex of poly-γ-glutamic acid (γ-PGA) and alum (PGA/alum) induced cross-protective immunity through a pandemic H1N1 vaccine against heterologous viruses in mice [47]. In this study, we report that vaccination with a high dose of a 3M2e–3HA2–NP chimeric protein (15 μg) provided marked protection against A/Puerto Rico/8/1934 (PR8) virus infections in mice. Furthermore, the use of PGA/alum allows for chimeric proteins to be spared and vaccine efficacy to be improved, comparable to high doses of chimeric-protein vaccinations in mice. These results suggest that the PGA/alum-adjuvanted chimeric protein may be a potential UIV candidate against influenza viruses.

## Materials and Methods

### Mice

C57BL/6 and BALB/c mice (six to eight weeks old, female) were purchased from Orient Bio Inc. (Republic of Korea). All mice were housed and maintained under specific pathogen-free (SPF) conditions in the Korea Research Institute of Bioscience and Biotechnology (KRIBB). All studies were reviewed and approved by the Institutional Animal Care and Use Committee (IACUC) at the KRIBB (KRIBB-AEC-17161 and KRIBB-AEC-16186). All animal experiments were performed according to the Guidelines for Animal Experiments of the KRIBB.

### Cells

Madin-Darby Canine Kidney (MDCK) cells were purchased from the American Type Culture Collection (ATCC) and maintained in Modified Eagle’s Medium (MEM; Corning, USA) containing 5% heat-inactivated fetal bovine serum (FBS; Gibco, USA), 100 U/ml penicillin, 100 mg/ml streptomycin (Gibco), and 1% MEM vitamin solution (Sigma, USA). Splenocytes from immunized mice were cultured in Roswell Park Memorial Institute (RPMI) 1640 medium (Gibco) supplemented with 10% heat-inactivated FBS, 100 U/ml penicillin, and 100 mg/ml streptomycin.

### Preparation of PGA/Alum

γ-PGA was purchased from BioLeaders (Korea), and PGA/alum was prepared by combining γ-PGA with Imject alum (Thermo Fisher, USA) in deionized distilled water (DDW) containing 0.9% saline as previously reported [47]. Briefly, 1 mg/ml of alum solution (200 ml, pH 6.5) was added dropwise to 1 mg/ml of γ-PGA solution (200 ml, pH 6.8) with constant stirring at 600 rpm. The combined PGA/alum was centrifuged at 15,000 ×*g* for 30 min at 4°C and resuspended in DDW containing 0.9% saline. The combined PGA/alum was stored at 4°C prior to use.

### Construction of Plasmids Expressing 3M2e, 3HA2, NP, and 3M2e–3HA2–NP (Chimeric) Proteins

The genes of the influenza A/Puerto Rico/8/34 (PR8) strain were used as a template for cloning 3M2e, 3HA2, and NP. The cDNA-encoding three tandem repeats of M2e (amino acid residues 2–24) were cloned by NheI and NotI into pET21a(+) (Novagen, USA). Each M2e sequence was linked by a 6 bp linker (TCTAGC). Three tandem repeats of HA2 (amino acid residues 420–474)-encoding cDNA were cloned by NheI and XhoI into pET21a(+). Each HA2 sequence was linked by a 6 bp linker (TCTAGC). NP (amino acid residues 2–498)-encoding cDNA was also cloned by NheI and XhoI into pET21a(+). To construct the plasmid-containing the 3M2e–3HA2–NP (chimeric) sequence, three tandem repeats of M2e, three tandem repeats of HA2, and NP were linked by G4S linkers to form the chimeric construct. The chimeric construct was cloned by NheI and NotI into pET21a(+).

### Expression and Purification of 3M2e,3HA2, NP, and 3M2e–3HA2–NP (Chimeric) Proteins

The constructs (3M2e, 3HA2, NP, and 3M2e–3HA2–NP) in pET21a(+) containing C-terminal 6×His-tag were transformed into *Escherichia coli* C43(DE3) by heat shock for 30 s at 42°C. The transformed cells were selected at 37°C on a Luria–Bertani (LB) broth agar plate containing 100 μg/ml of ampicillin (LPS Solution, Korea). A selected single colony was inoculated in 5 ml of LB broth with 100 μg/ml of ampicillin and incubated overnight in a shaking incubator at 200 rpm and 37°C. The overnight culture was transferred into 500 ml of fresh LB broth with 100 μg/ml of ampicillin and incubated in a shaking incubator at 200 rpm and 37°C until the optical density (OD) at 600 nm reached 0.3–0.6. Then, isopropyl β-D-1-thiogalactopyranoside (IPTG) was added to the cells at a final concentration of 1 mM to induce protein expression and then incubated overnight at 160 rpm and 20°C. After incubation, the cells were centrifuged at 12,000 ×*g* for 30 min at 4°C and stored overnight at –80°C. For the purification of proteins, the frozen cells were thawed and resuspended in binding buffer (50 mM NaH_2_PO_4_, 0.5 M NaCl, 20 mM imidazole, 1% Triton X-100, pH 8.0). The resuspended cells were disrupted by sonication on ice, and the soluble fraction was separated by centrifugation at 12,000 ×*g* for 30 min at 4°C. The soluble fraction was applied to a Ni–NTA column (Protein Ark, UK) using ÄKTA Explorer (GE Healthcare, USA). After loading the proteins, the column was washed with washing buffer (50 mM NaH_2_PO_4_, 0.5 M NaCl, 20 mM imidazole, pH 8.0), and proteins were eluted with elution buffer (50 mM NaH_2_PO_4_, 0.5 M NaCl, 0.5 M imidazole, pH 8.0). The eluted proteins were concentrated by an Amicon Ultra centrifugal filter (Millipore, USA), followed by size-exclusion chromatography using a HiLoad Superdex 200 column (GE Healthcare) with a size-exclusion buffer (50 mM NaH_2_PO_4_, 0.5 M NaCl, pH 7.4). The expected proteins were collected, and the buffer was changed with an Amicon Ultra centrifugal filter in phosphate-buffered saline (PBS). Then, purified proteins were concentrated with an Amicon Ultra centrifugal filter, and the protein concentration was determined using a BCA assay kit (Thermo Scientific).

### SDS-PAGE and Western Blot

Purified proteins (chimeric, 3M2e, 3HA2, and NP) were separated by 12–15% SDS-polyacrylamide gel electrophoresis and visualized by staining with Coomassie blue R-250 (LPS Solution, Korea). For western blot analysis, the proteins separated by 12–15% SDS-polyacrylamide gel were transferred to polyvinylidene difluoride (PVDF) membranes (Millipore). The membranes were blocked with 5% skim milk in PBS for 1 h at room temperature (RT). After blocking, the membranes were washed with PBST (PBS containing 0.1% Tween-20) and incubated with 1:2000 primary antibodies specific for influenza A H1N1 HA2 (Biorbyt, UK), influenza A M2 (Invitrogen, USA), influenza A NP (Southern Biotech, USA), and Penta·His (Qiagen, The Netherlands) overnight at 4°C. After washing with PBST, the membranes were incubated with 1:2000 horseradish peroxidase (HRP)-conjugated horse anti-mouse IgG or HRP-conjugated goat anti-rabbit IgG (Cell Signaling, USA) for 1h at RT. After being washed with PBST, protein bands were visualized by an enhanced chemiluminescence system (GE Healthcare).

### Preparation of Influenza A Viruses

Influenza A/Puerto Rico/8/34 (PR8; H1N1), A/California/04/09 (CA04; H1N1), and H3N2 carrying HA and NA genes from A/Hong Kong/1/68, as well as six internal genes from A/Puerto Rico/8/34, were provided by Dr. Young Ki Choi (Chungbuk National University, Cheongju, Korea). Influenza A viruses (PR8, CA04, and H3N2) were inoculated into 9–10-day-old SPF-embryonated chicken eggs and incubated for two days at 37°C. After chilling at 4°C overnight, the allantoic fluid containing influenza viruses was collected and centrifuged at 3,500 ×*g* for 10 min at 4°C. Then, the allantoic fluid was filtered through a 0.45 μm pore-size membrane filter (Millipore) and stored at –80°C until use. All virus experiments were performed under Biosafety Level 2+ (BSL2+) facilities and conditions.

### Immunization and Virus Challenge

C57BL/6 mice (6–8 weeks old, female) were intramuscularly (i.m.) immunized with 1–15 μg of chimeric protein in the presence or absence of 400 μg of γ-PGA, 400 μg of alum, and 800 μg of PGA/alum on days 0, 14, and 28. Two weeks after the last immunization, the sera from the immunized mice were collected, and the immunized mice were intranasally (i.n.) challenged with a lethal dose (LD) of PR8 (10 LD_50_), CA04 (20 LD_50_), or H3N2 (10 LD_50_). Body weight and survival rate were monitored for 14 days. Mice that had lost more than 25% of their initial body weight were considered dead and were humanely euthanized.

### Enzyme-Linked Immunosorbent Assay (ELISA)

The level of influenza A virus antigen-specific IgG antibody in the sera from the immunized mice was measured using ELISA. ELISA plates (Nunc, USA) were coated overnight at 4°C with 2 μg/ml of 3M2e, 3HA2, and NP in PBS. Antigen-coated plates were washed twice with PBST and blocked with 5% skim milk in PBS for 1 h at RT. After washing twice, diluted samples in blocking buffer were added to the plates and 3-fold serially diluted in the plates. After incubation for 2 h, the plates were washed twice, and 1:5000 secondary detection antibodies (horseradish-peroxidase-conjugated goat anti-mouse IgG) were added to the plates. After incubation for 1 h at RT, plates were washed twice, and 100 μl of 3,3’,5,5’-tetramethylbenzidine (TMB) was added for the development of the reactions. Reactions were stopped by the addition of 50 μl of 1 M sulfuric acid. OD was measured at a wavelength of 450 nm using a microreader.

### Flow Cytometry

C57BL/6 mice were i.m. vaccinated with chimeric protein (1–15 μg) alone or mixed with 400 μg of γ-PGA, 400 μg of alum, and 800 μg of PGA/alum on days 0, 14, and 28. Two weeks after the last vaccination, the mice were sacrificed, and splenocytes were obtained from the mice. Splenocytes (1 × 10^6^ cells/100 μl) were blocked with 1 μl of anti-CD16/32 monoclonal antibody (mAb; BD Biosciences, USA) and surface-stained with an antibody mixture containing 1 μl of anti-CD3e-PerCP (BD Biosciences), anti-CD4-FITC (BD Biosciences), and anti-CD8-allophycocyanin (BD Biosciences). After incubation for 30 min at 4°C, surface-stained cells were washed twice with PBS. Then, cells were fixed and permeabilized with a BD Cytofix/Cytoperm Kit (BD Biosciences) according to the manufacturer’s instructions. Fixed and permeabilized cells were stained with 1 μl of anti-IFN-γ-PE (BD Biosciences) for 30 min at 4°C, and cells were detected by FACSVerse or FACS Calibur flow cytometry (BD Biosciences). FlowJo software (Tree Star, USA) was used for data analysis.

### Antibody-Dependent NK Cell Activation

The 3HA2 (2 μg/ml, stalk region) protein was coated onto the ELISA plates. Coated plates were washed twice with PBS and incubated with heat-inactivated serum (1 h, 56°C) of the immunized mice for 2 h at 37°C. Then, the unbound antibodies in serum were washed twice with PBS. For NK cell preparation, the spleen was isolated from naïve C57BL/6 mice, and splenocytes were obtained from the spleen. NK cells in the splenocytes were purified by an NK cell isolation kit (Miltenyi Biotec, Germany) according to the manufacturer’s instructions. Isolated NK cells (1 × 10^5^ cells/well) were added to the wells and incubated with anti-CD107a-PE (BD Biosciences) in the presence of a protein transport inhibitor cocktail (eBioscience, USA) for 6 h at 37°C. After incubation, NK cells were harvested, fixed, and permeabilized by a BD Cytofix/Cytoperm Kit (BD Biosciences) according to the manufacturer’s instructions. Then, NK cells were stained with anti-IFN-γ-allophycocyanin (BD Biosciences) and detected by FACSVerse or FACS Calibur flow cytometry (BD Biosciences).

### Antibody-Dependent Cellular-Cytotoxicity (ADCC) Assay

Influenza A viruses (PR8, CA04 or H3N2)-infected MDCK cells (MOI = 1) were harvested and added to U-bottom 96-well plates at 1 × 10^4^ cells/well. Virus-infected MDCK cells were incubated with heat-inactivated serum (1 h, 56°C) and naïve NK cells (1 × 10^4^ cells/well) for 4 h at 37°C. Cytotoxicity of the NK cells was measured by lactate dehydrogenase (LDH) in culture supernatants using a CytoTox 96 non-radioactive cytotoxicity assay (Promega, USA) according to the manufacturer’s instructions.

### Enzyme-Linked Immunospot (ELISPOT)

ELISPOT assay was performed with a mouse IFN-γ ELISPOT Kit (BD Biosciences) according to the manufacturer’s instructions. Briefly, ELISPOT plates were coated with purified anti-IFN-γ at 4°C overnight. The splenocytes obtained from the immunized mice were plated at 5 × 10^5^ cells/well on the anti-IFN-γ-coated ELISPOT plates and stimulated with 2000 TCID_50_ of UV-inactivated influenza A viruses (PR8, CA04 or H3N2). After three days, the spot-forming units (SFUs) of IFN-γ-producing cells were developed by AEC substrate (BD Biosciences).

### Hemagglutination-Inhibition (HI) Assay

HI titers of the sera against influenza A viruses (PR8, CA04, and H3N2) were determined as described previously [48]. Briefly, the sera from the immunized mice were treated with a receptor-destroying enzyme (RDE; Hardy Diagnostics, USA) and serially 2-fold diluted with PBS in U-bottom 96-well plates. The diluted sera were incubated with 4 HA units/25 μl of influenza A viruses (PR8, CA04, or H3N2) for 30 min at RT. After incubation, 0.7% turkey red blood cells (RBCs) were added to the sera mixed with the virus and incubated for 30 min at RT. HI titers were determined by the highest dilution that prevents hemagglutination.

### Virus Titers in Lungs

C57BL/6 mice (6–8 weeks old, female) were i.m. immunized three times with a chimeric protein (1–15 μg) in the presence or absence of 400 μg of alum, and 800 μg of PGA/alum at 2-week intervals. Two weeks after the last vaccination, influenza A viruses (PR8, CA04 or H3N2) were challenged in the mice, and lung samples from the mice were obtained at seven days post-infection (dpi). Lung samples were homogenized in MEM containing 100 U/ml penicillin, 100 mg/ml streptomycin, and 1% MEM vitamin solution and were centrifuged at 12,000 ×*g* for 15 min at 4°C. Supernatants were used for virus titration. The virus titers were calculated based on Reed and Muench method [49] and expressed as log_1o_ 50% tissue culture infectious dose (TCID_50_)/ml.

### Immunohistochemistry

Immunohistochemistry (IHC) was performed as described previously [48, 50]. Briefly, the immunized mice (C57BL/6, female) were i.n. challenged with 10 LD_50_ of PR8, 20 LD_50_ of CA04, or 10 LD_50_ of H3N2. Seven days after virus infection, the mice were sacrificed and lung tissues from the mice were obtained, fixed with 10% neutral-buffered formalin, and embedded in paraffin. Formalin-fixed, paraffin-embedded tissue was cut into 4 μm sections and mounted onto glass slides. Tissue slides were blocked with 10% normal goat serum in PBS for 1 h at RT, washed with PBS containing 0.1% Triton X-100 (PBSX), and stained with anti-influenza A NP antibody (Southern Biotech, USA) for 2 h at RT. After washing with PBSX, slides were stained with Alexa Fluor 488-conjugated goat anti-mouse IgG (Invitrogen) for 2 h at RT, followed by staining with 10 μg/ml of 4´,6-diamidino-2-phenylindole (DAPI; Sigma, USA) for 10 min at RT. Then, the stained tissue slides were examined using an LSM5 Pascal confocal fluorescence microscope (Carl Zeiss, Germany).

### In Vivo Protection Assay

To identify whether immune serum from the immunized mice would cross-protect the mice against influenza A viruses (PR8, CA04, or H3N2), in vivo protection assay was carried out as previously described [14, 51]. Briefly, heat-inactivated (56°C, 1 h) immunized serum was mixed with 2 LD_50_ of PR8, 3 LD_50_ of CA04, or 2 LD_50_ of H3N2 and incubated for 30 min at RT. Then, naïve BALB/c mice were i.n. infected with the mixture of sera and virus. Body weight and survival rate were monitored for 14 days.

### In Vivo Depletion of Immune Cells

Mice (C57BL/6, female) were immunized three times at 2-week intervals. The mice were infected with 10 LD_50_ of PR8 on day 42 after initial immunization. To deplete immune cells, the mice were intraperitoneally injected with 450 μg of anti-CD4 (rat IgG2b; Bio X Cell, USA), anti-CD8 (rat IgG2b; Bio X Cell), anti-NK1.1 (mouse IgG2a; Bio X Cell), or isotype control antibody (mouse IgG2a; Bio X Cell) on days 39, 41, and 43. After virus infections, body weight and survival rate were monitored for 14 days.

### Statistical Analysis

Statistical analysis was performed using GraphPad Prism version 8 (GraphPad Software, USA). Data are presented as the mean ± standard error of the mean (SEM). Statistically significant difference among multiple groups was assessed using one-way ANOVA/Bonferroni. *P*-values less than 0.05 indicates statistical significance.

## Results

### Recombinant 3M2e–3HA2–NP (Chimeric) Protein Expressed and Purified in *E. coli*

**Fig. 1:**
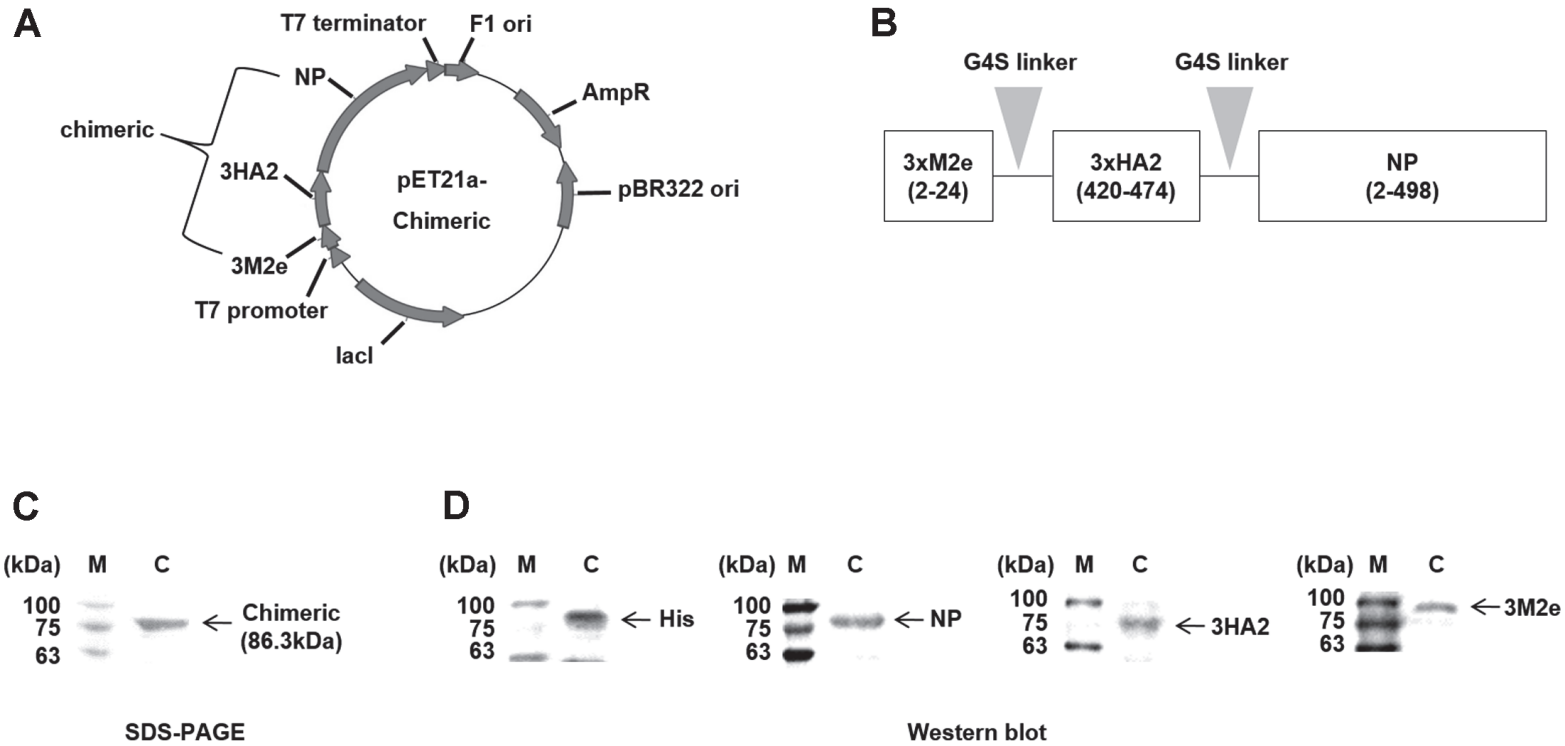
Vaccine construction and confirmation of purified 3M2e–3HA2–NP (chimeric) protein using the *E. coli* C43(DE3) expression system. (**A**) Vector map of the pET21a(+) harboring chimeric protein sequence composed of three tandem repeats of M2e, three tandem repeats of HA2, and NP. (**B**) Schematic diagram of chimeric protein consisting of components (3×M2e, 3×HA2, and NP) linked by a G4S linker (gray arrowhead). (**C, D**) Chimeric protein expressed in *E. coli* C43(DE3) and purified by His-tag affinity chromatography. Expression was confirmed by (**C**) SDS-PAGE or (**D**) western blot (anti-His, anti-NP, anti-HA2, or anti-M2 (containing M2e sequence) antibodies). M, marker; C, chimeric protein.

The chimeric protein was composed of 3×M2e, 3×HA2, and NP. Amino acid residues 2–24 of M2e, 420–474 of HA2, and 2–498 of NP were expressed simultaneously, and each conserved domain (M2e, HA2, and NP) was connected to a G4S linker in the chimeric protein. The conserved domains of 3M2e, 3HA2, and NP from PR8 virus were synthesized and inserted into a pET21a(+) vector to express the chimeric protein as a vaccine antigen (Figs. 1A-1B). The expressed chimeric protein was detected in SDS-PAGE, and its purity was >90% (Fig. 1C). The expression of the purified chimeric protein was confirmed by western blot using anti-His, anti-NP, anti-HA2, and anti-M2e antibodies (Fig. 1D).

### Immunization of Chimeric Protein Induces Dose-Dependent Protection Against PR8 Virus 

**Fig. 2:**
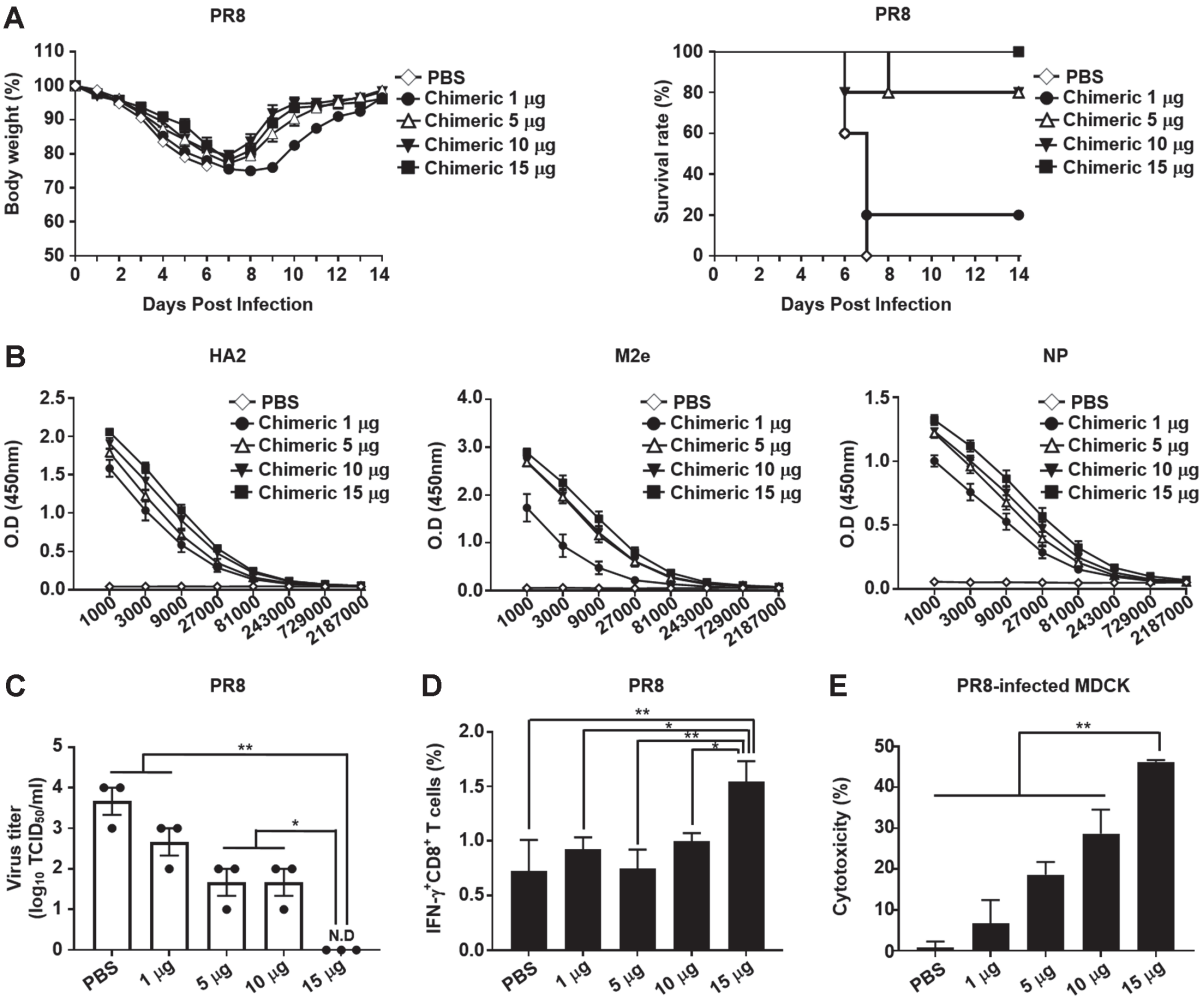
Vaccination with the chimeric protein induces protection efficacy against the PR8 virus in mice. C57BL/6 mice (*n* = 5 per group) i.m. immunized with 0, 1, 5, 10, and 15 μg of chimeric protein three times at 2-week intervals. (**A**) Two weeks after the last vaccination, mice were challenged with 10 LD_50_ of PR8 in vaccinated groups. Body weight and survival rate were measured for two weeks. (**B**) Sera were collected two weeks after the last immunization. 3HA2-, 3M2e-, or NP-specific IgG in sera determined by ELISA using purified 3HA2, 3M2e, or NP as coating antigens. (**C**) Viral titers in lung homogenates at 7 dpi were determined by TCID_50_ assay (N.D, not detected). (**D**) Splenocytes from immunized mice were stimulated by 2000 TCID_50_ of UV-inactivated PR8 virus. Then, virus-specific IFN-γ + CD8^+^ T cells were detected by flow cytometry. (**E**) Cytotoxicity of NK cells was measured by lactate dehydrogenase (LDH) assay in PR8-infected MDCK cells (MOI = 1). Data are representative of three independent experiments and expressed as the mean ± SEM. Statistically significant differences were determined using one-way ANOVA/Bonferroni; *p < 0.05, **p < 0.01.

To evaluate the efficacy of the chimeric protein, C57BL/6 mice were i.m. immunized three times with chimeric protein (0, 1, 5, 10, and 15 μg) at 2-week intervals. Fourteen days after the last immunization, the chimeric protein-immunized mice were challenged with 10 LD_50_ of the PR8 virus. After PR8 virus infection, body weight decreased for eight days in the chimeric groups (1, 5, 10, and 15 μg), and the entire PBS group was dead on day 7. Body weight recovered to the initial body weight dose-dependently in the chimeric groups on day 14, but only the 15 μg of chimeric group showed 100% survival. The other groups had 80% protection (5 and 10 μg of chimeric groups) and 20% protection (1 μg of chimeric group) against PR8 virus (Fig. 2A).

To evaluate the antibody response of the chimeric protein, conserved domains (3HA2, 3M2e, and NP) were purified and the expression of the proteins was confirmed by SDS-PAGE (Figs. S1A-S1C). The purified proteins were coated onto the plates and diluted sera were incubated for 1 h. IgG antibody titers were measured by ELISA. Results showed that the level of 3HA2-, 3M2e-, or NP-specific IgG was increased dose-dependently, but antibody titers were not significantly different among groups (0, 1, 5, 10, and 15 μg of chimeric protein; Fig. 2B). To better understand the efficacy of chimeric protein, viral clearance was examined in lungs from the immunized mice. The mice were i.m. immunized with chimeric protein (0, 1, 5, 10, and 15 μg), and challenged with PR8 virus. Lungs were collected at 7 dpi and viral titers in the lung homogenates were measured by TCID_50_. The viral titers were significantly reduced dose-dependently and completely cleared in the 15 μg of chimeric group at 7 dpi (Fig. 2C).

To detect the involvement of CD8^+^ T cells in chimeric protein vaccination, the frequency of IFN-γ^+^ CD8^+^ T cells was detected by flow cytometry to observe CTL response. Analysis of flow cytometry revealed that the 15 μg of chimeric group induced significantly higher frequency of IFN-γ^+^ CD8^+^ T cells compared with the other groups (0, 1, 5, and 10 μg of chimeric groups; Fig. 2D). Subsequently, we investigated whether antibodies from the sera-protected mice neutralized the PR8 virus. In vivo protection assay showed that sera from the immunized mice could provide partial protection against the PR8 virus, but did not induce an HI-positive titer against this virus (Figs. S2A and S2B). Non-neutralizing antibody is related to the ADCC response by NK cells [27, 52]. The ADCC response by antibodies and NK cells is an important mechanism for cross-protection against influenza viruses [53, 54]. To detect the cytotoxicity of NK cells against the PR8 virus, PR8-infected MDCK cells were co-cultured with NK cells in the presence of antibodies from the immunized mice. Then, the cytotoxicity of NK cells was measured using LDH assay. As shown in Fig. 2E, the cytotoxicity of the chimeric group (15 μg) was significantly different from that of the other groups (0, 1, 5, and 10 μg of chimeric groups). The 15 μg of chimeric group showed the highest cytotoxicity (46.24 ± 0.46%) compared with that of the other groups (0.74 ± 1.53% for PBS, 6.77 ± 5.68%for 1 μg of chimeric, 18.66 ± 3.09% for 5 μg of chimeric, and 28.67 ± 5.91% for 10 μg of chimeric) in PR8-infected MDCK cells. These data indicate that the chimeric protein induces antibody responses, ADCC by NK cells, and CD8^+^ T cell activation against the PR8 virus.

### PGA/Alum Enhances Cross-Reactivity of Chimeric Protein Against Influenza A Viruses

**Fig. 3:**
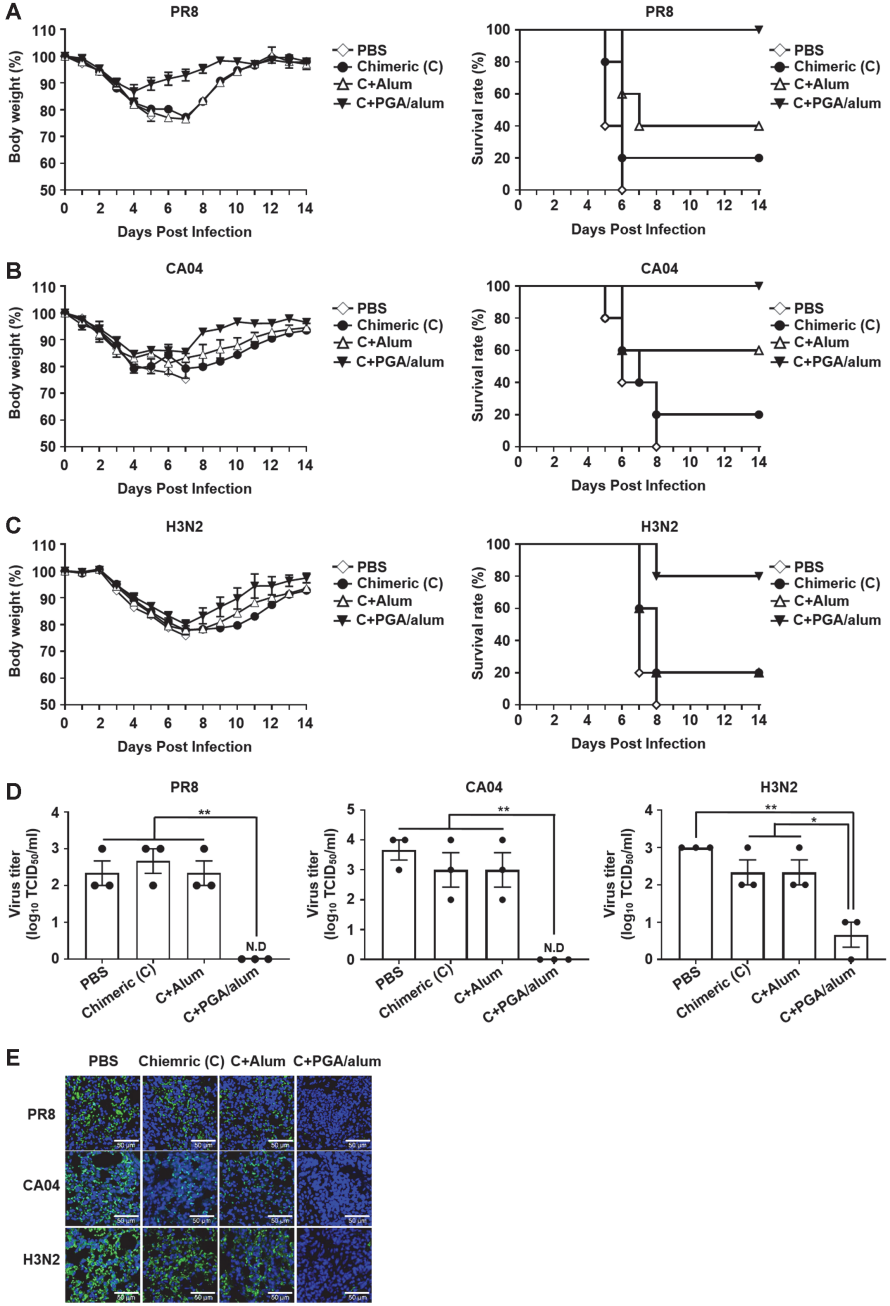
Cross-reactivity of the chimeric protein against influenza A viruses is enhanced by PGA/alum. C57BL/6 mice (*n* = 5 per group) were i.m. vaccinated with (**A**, **B**) 1 or (**C**) 5 μg of chimeric protein with alum or PGA/alum three times at 2-week intervals. Two weeks after the last vaccination, the mice were challenged with (**A**) 10 LD_50_ of PR8, (**B**) 20 LD_50_ of CA04, or (**C**) 10 LD_50_ of H3N2. (**A**–**C**) Body weight and survival rate were measured for two weeks. (**D**) Lungs were aseptically collected from vaccinated mice at 7 dpi, and the viral titer in lung homogenates was determined by TCID_50_ assay (N.D, not detected). (**E**) Influenza viruses in lung tissues from immunized mice were detected by confocal microscopy using anti-NP antibody and Alexa Fluor 488-conjugated goat anti-mouse IgG after influenza A virus (PR8, CA04, or H3N2) challenge. Scale bar, 50 μm. Data are expressed as the mean ± SEM. Statistical significance was determined by one-way ANOVA/Bonferroni; *p < 0.05, **p < 0.01.

To spare the dose and enhance the vaccine efficacy of chimeric protein, PGA/alum was used as an adjuvant. Mice were vaccinated with 1 μg of chimeric protein with PGA/alum. Fig. 3A shows that PGA/alum enhanced the efficacy of the chimeric protein compared with the chimeric protein alone after homologous PR8 virus infection. Mice immunized with PGA/alum-adjuvanted chimeric protein showed less severe reduction of body weight than other groups (PBS, chimeric, and chimeric with alum groups). PGA/alum-adjuvanted chimeric conferred 100%protection, but chimeric alone and chimeric with alum provided only 20% and 40% protection, respectively, to the mice. The PBS group had 0% protection. Moreover, the effect of γ-PGA as an adjuvant was investigated with chimeric protein to compare it with PGA/alum. As shown in Figs. S3A-S3C, γ-PGA provided weak adjuvant effects on chimeric protein compared to PGA/alum against PR8 virus.

Intrasubtypic protection was also investigated in 1 μg of chimeric with PGA/alum-vaccinated mice after intrasubtypic CA04 (H1N1) virus challenge. The PGA/alum-adjuvanted chimeric group showed 100%protection in mice, but chimeric alone and chimeric with alum conferred 20% and 60% survival, respectively. The entire PBS group was dead after virus infection (Fig. 3B). Heterosubtypic cross-protection of 5 μg of chimeric with PGA/alum was also examined after H3N2 virus infection. The results indicated that PGA/alum-adjuvanted chimeric provided 80% protection to the mice, and chimeric alone and chimeric with alum protected 20% of the vaccinated mice after virus infection. The PBS group did not gain any protection (Fig. 3C). These results suggest that PGA/alum improves the efficacy of the chimeric protein and enhances its cross-protection against homo- and heterosubtypic influenza viruses.

To better ascertain the efficacy of PGA/alum-adjuvanted chimeric protein, viral clearance was investigated in mice. The mice were i.m. immunized with chimeric protein (1 or 5 μg) in the presence or absence of alum or PGA/alum three times, and the vaccinated mice were challenged with influenza A viruses (PR8, CA04, and H3N2). Then, lungs were isolated from the mice at 7 dpi, and viral titers were measured in lung homogenates. As shown in Fig. 3D, the viral titer level was significantly reduced in PGA/alum-adjuvanted chimeric compared with that in PBS, chimeric, and chimeric with alum groups at 7 dpi. To confirm the reduction of viral titer in the lungs, the NP protein of influenza A viruses was detected by immunofluorescence confocal microscopy. As expected, the NP protein was rarely observed in the chimeric with PGA/alum, but other groups (PBS, chimeric, and chimeric with alum groups) clearly exhibited the presence of influenza A viruses under confocal microscopy (Fig. 3E). These results reveal that PGA/alum increases the efficacy of the chimeric protein to clear viruses in the lungs after influenza A virus infection.

### PGA/Alum Increases Virus-Specific Humoral and Cellular Immune Responses of Chimeric Protein 

**Fig. 4:**
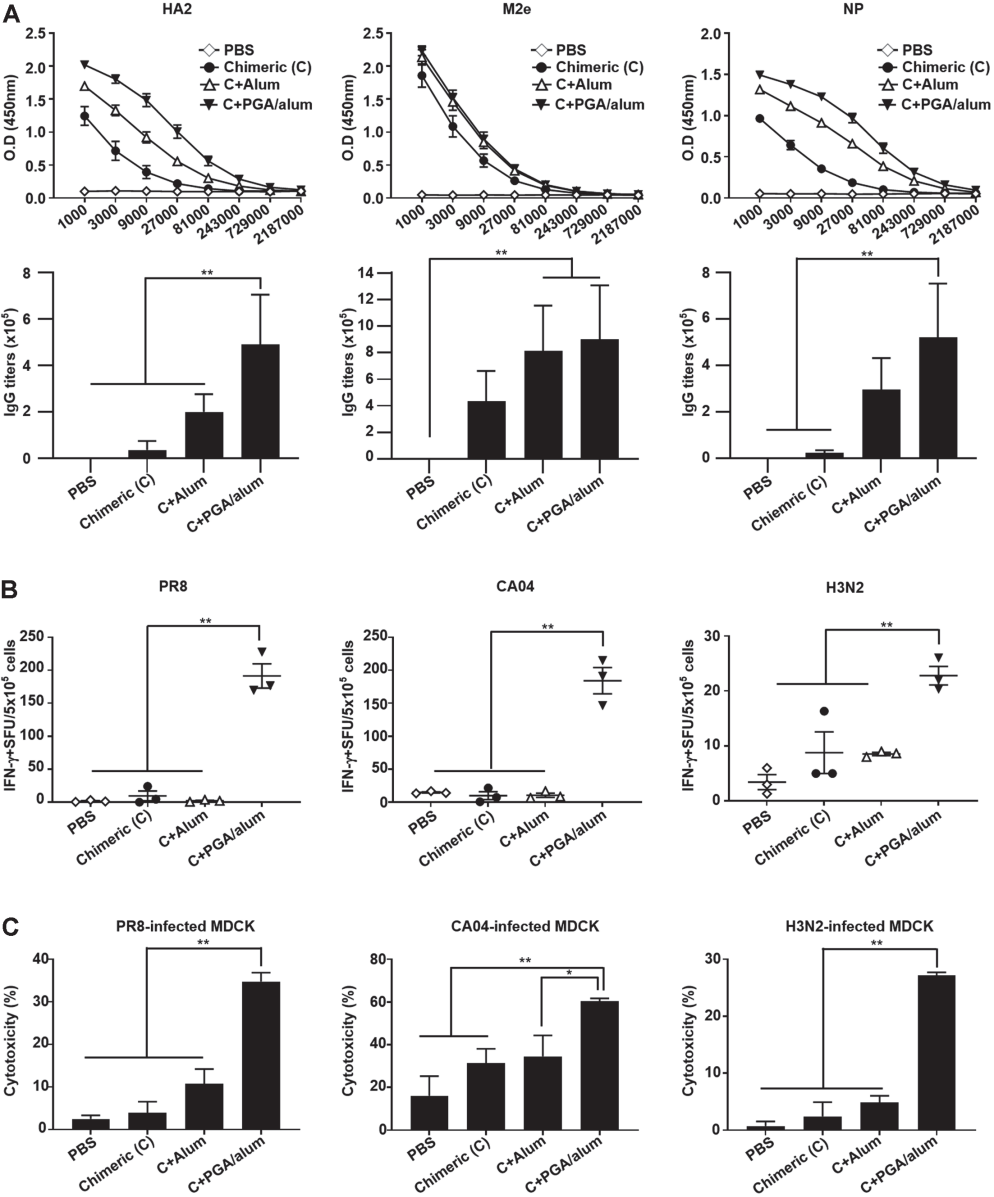
PGA/alum enhances humoral and cellular immune responses of the chimeric protein against influenza A viruses. C57BL/6 (*n* = 5 per group) vaccinated i.m. three times with 1 or 5 μg of chimeric protein in the presence or absence of alum and PGA/alum. (**A**) Two weeks after the last vaccination, sera were collected from immunized mice. Conserved domains (3HA2, 3M2e, and NP) of the chimeric protein were coated in plates and diluted-sera of immunized mice were incubated for 1 h. IgG antibody titers were measured by ELISA assay. (**B**) Splenocytes from immunized mice were stimulated by 2000 TCID_50_ of UV-inactivated viruses (PR8, CA04, and H3N2). Then, virus-specific T cells were detected by ELISPOT. (**C**) NK cell cytotoxicity against influenza A viruses (PR8, CA04, and H3N2) was measured by LDH assay. Data are shown as the mean ± SEM. Statistically significant differences were determined by one-way ANOVA/Bonferroni; *p < 0.05, **p < 0.01.

The humoral and cellular immune responses of the PGA/alum-adjuvanted chimeric protein were investigated to better understand the effect of PGA/alum on the chimeric protein. To evaluate the antibody responses of the PGA/alum-adjuvanted chimeric protein, IgG antibody titers against 3HA, 3M2e, and NP of the chimeric protein were measured by ELISA. As shown in Fig. 4A, 3HA2, 3M2e, and NP-specific IgG titers were highest in the PGA/alum-adjuvanted chimeric group compared with those in the other groups (PBS, chimeric, and chimeric with alum groups). However, the antibodies provided partial protection against influenza A viruses and could not induce an HI-positive titer against the viruses (PR8, CA04, and H3N2) in the PGA/alum-adjuvanted chimeric group (Figs. S4A and S4B).

To further understand the mechanism of PGA/alum-adjuvanted chimeric protein, we examined the binding affinity of IgG antibodies against influenza A viruses, NK cell activation, and ADCC activity by NK cells. As shown in Fig. S5A, the binding affinity of IgG antibodies was highest in the PGA/alum-adjuvanted chimeric group and was significantly different from the other groups (PBS, chimeric, and chimeric with alum). Moreover, the frequency of the activated NK cells (CD107a^+^ IFN-γ^+^ NK cells) was also highest in PGA/alum-adjuvanted chimeric group compared with that in the other groups (PBS, chimeric, and chimeric with alum groups) by flow cytometry analysis (Fig. S5B).

To ascertain the cross-reactivity of T cells, mice were vaccinated with chimeric, chimeric with alum, and PGA/alum-adjuvanted chimeric. Then, splenocytes from the immunized mice were harvested and activated with three different UV-inactivated influenza A viruses (PR8, CA04, or H3N2). As shown in Fig. 4B, ELISPOT analysis revealed that the PGA/alum-adjuvanted chimeric produced more PR8-, CA04-, and H3N2-specific IFN-γ-secreting cells compared with the other groups (PBS, chimeric, and chimeric with alum groups). To further identify cross-reactive CD8^+^ T cells, IFN-γ secreting CD8^+^ T cells were detected by flow cytometry after treatment of UV-inactivated influenza A viruses (PR8, CA04, or H3N2). Results showed that the frequency of IFN-γ^+^ CD8^+^ T cells was significantly increased by the PGA/alum-adjuvanted chimeric compared with that in the other groups (Figs. S6A- S6C).

For the ADCC assay, the cytotoxicity of the NK cells was examined using an LDH assay. In PR8-infected MDCK cells, the PGA/alum-adjuvanted chimeric group had the highest cytolysis activity (34.74 ± 2.11%) compared with that of other groups (2.47 ± 0.87% for PBS, 3.92 ± 2.64% for chimeric, and 10.73 ± 3.50% for chimeric with alum groups). Similarly, the cytotoxicity of the PGA/alum-adjuvanted chimeric group was significantly higher (60.33 ± 1.40%) than that of PBS (16.13 ± 9.18%), chimeric (31.29 ± 6.78%), and chimeric with alum (34.56 ± 9.80%) groups in intrasubtypic CA04-infected MDCK cells. Moreover, the PGA/alum-adjuvanted chimeric group had significantly higher cytotoxicity (27.16 ± 0.56%) than that of PBS (0.72 ± 0.82%), chimeric (2.41 ± 2.51%) and chimeric with alum (4.86 ± 1.17%) groups in heterosubtypic H3N2-infected MDCK cells (Fig. 4C).

Taken together, these findings demonstrate that PGA/alum enhances the cross-protection of the chimeric protein against influenza A viruses through humoral and cellular immunities.

### Depletion of NK and T Cells Blocks Protection of PGA/Alum-Adjuvanted Chimeric Protein Against PR8 Virus

**Fig. 5:**
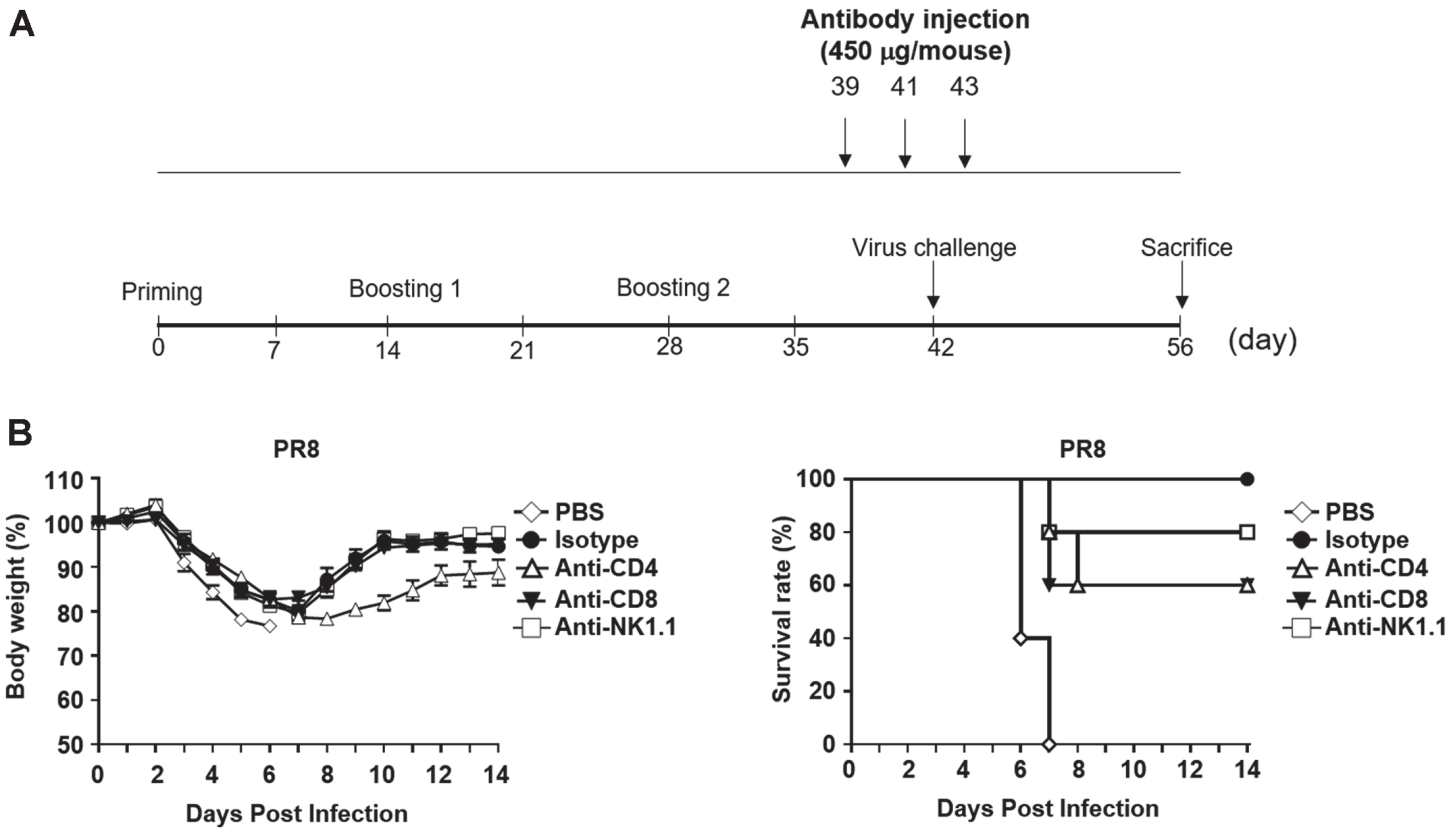
Protection of the PGA/alum-adjuvanted chimeric protein against the PR8 virus was impaired in the absence of NK and T cells. (**A**) Experimental schedule of vaccination and antibody depletion. (**B**) C57BL/6 mice (*n* = 5 per group) were i.m. immunized three times with 1 μg of chimeric protein in the presence of 800 μg of PGA/alum on days 0, 14, and 28. On day 42, mice were challenged with 10 LD_50_ of the PR8 virus. For depletion of CD4^+^ T, CD8^+^ T, or NK cells, 450 μg of antibodies (anti-CD4, anti-CD8, or anti-NK1.1) was injected on days 39, 41, and 43. Body weight and survival rate were monitored for two weeks after the virus challenge.

To confirm whether the protection efficacy of mice vaccinated with PGA/alum-adjuvanted chimeric was mediated by CD4^+^ T, CD8^+^ T, and NK cells, C57BL/6 mice were i.m. immunized three times in the presence of 800 μg of PGA/alum. Two weeks after the last vaccination, the mice were challenged with 10 LD_50_ of the PR8 virus. For the depletion of CD4^+^ T, CD8^+^ T, or NK cells, 450 μg of the antibody was injected intraperitoneally in the mice (Fig. 5A). As shown in Fig. 5B, the depletion of CD4, anti-CD8, or anti-NK1.1 reduced the percentage of survival compared with the isotype control (100% survival rate) in the vaccinated mice after the PR8 challenge. The CD4^+^ T or CD8^+^ T cell-depleted group had 60% protection, and NK cell-depleted mice were 80% protected after the PR8 challenge. The entire PBS group was dead (0% survival rate). Taken together, our results demonstrate that CD4^+^ T, CD8^+^ T, and NK cells play major roles in protection against influenza virus in mice vaccinated with the PGA/alum-adjuvanted chimeric protein.

## Discussion

To produce an influenza virus vaccine, circulating virus strains are predicted, and virus vaccines are reformulated every year to induce immune responses toward those virus strains. However, antigenic mismatch between the circulating virus strains and vaccine antigens is a limitation of current influenza virus vaccines. In this study, the 3M2e–3HA–NP (chimeric) protein was constructed with conserved domains derived from the PR8 virus and vaccinated into mice. A PGA/alum adjuvant was also used to spare the dose and improve the efficacy of the chimeric protein. Our findings indicate that the PGA/alum-adjuvanted chimeric protein confers both homologous and heterologous protection against influenza A viruses by inducing virus-specific humoral and cellular immunity.

Accumulating reports indicate that HA2 might be a major target for broad protection against influenza A viruses because HA2 can induce broadly active anti-hemagglutinin antibodies against influenza A viruses [16, 55, 56]. Moreover, M2e has been widely used as a candidate of recombinant vaccines for cross-protection against influenza A viruses [29]. M2e is limited in inducing a strong immune response because it is too small to have high immunogenicity. M2e only promotes weak and transient immune responses against influenza viruses [57-59]. However, virus-like particles containing M2e can induce cross-protection, and a mucosal adjuvant enhances the immunogenicity of M2e against influenza A viruses [51, 60]. In addition, recombinant proteins NP-M2e or HA2-M2e elicit heterologous protection against influenza viruses by promoting robust immune responses [17, 38]. Because a current trend in the design of a universal influenza vaccine is the construction of recombinant proteins targeting various conserved epitopes of viral proteins (M2e, HA2, NP), we constructed and used chimeric protein (3M2e-3HA2-NP) as a universal vaccine antigen. Recently, various studies have been undertaken to check the cross-reactive vaccine effect of influenza chimeric proteins (HA2 and M2e) [44-46]. However, the cross-reactivity of adjuvanted chimeric protein 3M2e-3HA2-NP has never been investigated. In this study, we focused on the adjuvant effect of 3M2e-3HA2-NP to enhance the antigen dose-sparing effects as well as cross-protection of chimeric protein vaccine antigen.

The chimeric protein (15 μg) could induce 100% protection against the PR8 virus, and the virus in the lungs was cleared at 7 dpi, which means that the chimeric protein induced sufficient protection against the PR8 virus. In the antibody responses, the antibody level was dose-dependently increased against 3HA2, 3M2e, and NP proteins. The antibodies produced in response to 15 μg of chimeric protein could provide superior protection (60%) for mice compared with that of other groups (0 μg for 0%; 1, 5, and 10 μg for 20%) (Fig. S2A) even though the level of IgG in response to 15 μg of the chimeric protein was not significantly different from that of other groups (0, 1, 5, and 10 μg of chimeric protein). Thus, the antibody response induced by the chimeric protein could be involved in protection, and relevantly, the recombinant protein could promote an antibody response and protect mice against influenza viruses [61, 62]. In addition to the antibody response, 15 μg of the chimeric protein induced significantly higher IFN-γ^+^ CD8^+^ T cells compared with the low doses (0, 1, 5, and 10 μg) of the chimeric protein groups. The induction of IFN-γ^+^ CD8^+^ T cells by the chimeric protein is associated with the nucleoprotein of influenza virus that contains CD8^+^ T cell epitopes [40, 41]. Therefore, we propose that 15 μg of the chimeric protein induces sufficient immune responses and protection against the PR8 virus, which is mediated by both humoral and cellular immunity.

Recently, ADCC was reported to provide proper protection against various viruses, such as the influenza, human-immunodeficiency, and Epstein–Barr viruses [27, 63, 64]. Previous reports demonstrated that HA2 could induce ADCC-mediating antibodies [53, 65]. Consistent with these reports, the cytotoxicity of NK cells induced by the chimeric protein was dose-dependently increased and was significantly higher in the 15 μg of chimeric protein group than in the other groups (0, 1, 5, and 10 μg of chimeric groups). Thus, our findings suggest that the chimeric protein induced antibodies that could activate ADCC by NK cells. We also measured the level of IgG antibodies against 3HA2, 3M2e, and NP of the influenza A virus after vaccination with the chimeric protein in the presence or absence of adjuvants. The level of antibodies against 3HA2, 3M2e, and NP was enhanced by PGA/alum compared with chimeric alone or chimeric with alum. As expected, the antibodies were not positive in the HI assay because of the lack of the HA1 domain in the chimeric protein (Figs. S2B and S4B). Thus, we investigated the ADCC response by NK cells to identify whether the HA2-mediated antibody could be involved in ADCC. Surprisingly, the PGA/alum-adjuvanted chimeric protein substantially induced cytotoxicity against the virus-infected target cells and it was significantly higher than that of other groups in the ADCC assay. Taken together, we propose that the PGA/alum-adjuvanted chimeric protein promotes ADCC response against viral infection.

A crucial drawback of current influenza vaccines is that they will not prevent another pandemic in the future because they are strain-specific, not inducing cross-protection against other influenza viruses. It was reported that CD8^+^ T cells are critical factors for cross-protection [14, 39]. In this study, we investigated whether IFN-γ^+^ CD8^+^ T cells were significantly increased after vaccination with the PGA/alum-adjuvanted chimeric protein. Interestingly, PGA/alum significantly enhanced the induction of IFN-γ^+^ CD8^+^ T cells by low doses (1 or 5 μg) of the chimeric protein compared with chimeric alone or alum-adjuvanted chimeric protein. Our findings suggest that PGA/alum increases induction of IFN-γ^+^ CD8^+^ T cells in response to the chimeric protein, which is involved in cross-protection. Furthermore, our results showed that 1 μg of the chimeric protein with PGA/alum could confer similar protection against the PR8 virus compared with 15 μg of the chimeric protein. Thus, we propose that PGA/alum could be a suitable influenza vaccine adjuvant for cross-protection.

CD4^+^ T cells induce antibody production by B cells to promote viral clearance in influenza virus infection [66-68] and CD8^+^ T cells play a critical role in cross-protection against multiple influenza viruses [14, 39]. NK cell-mediated ADCC is also important for cross-protection against influenza A viruses [27]. Thus, depletion of these immune cells (CD4^+^ T, CD8^+^ T, or NK cells) may impair cross-protection against influenza viruses. In the present study, we depleted immune cells to confirm the effects of CD4^+^ T, CD8^+^ T, or NK cells in PGA/alum-adjuvanted chimeric group. As expected, the survival rate was decreased after treatment of anti-CD4, anti-CD8, or anti-NK1.1 antibodies in PGA/alum-adjuvanted chimeric group (Fig. 5B), which means CD4^+^ T, CD8^+^ T, or NK cells are involved in the protective effects of PGA/alum-adjuvanted chimeric protein.

Alum or γ-PGA-adjuvanted chimeric protein provided partial protection to mice (Figs. 3A-3C and Figs S3A-S3C). However, PGA/alum-adjuvanted chimeric protein conferred higher protection using the same chimeric dose compared with other adjuvants (alum or γ-PGA). Previously, we used an influenza split vaccine antigen kindly provided by Green Cross Corp. to investigate the adjuvant effect of PGA/alum. In this study, we used a chimeric recombinant protein (3M2e-3HA2-NP) as a vaccine antigen adjuvanted with PGA/alum to investigate heterosubtypic cross-reactivity. Mice vaccinated with chimeric protein only (15 μg) induced 100% protection against homologous virus (PR8; H1N1). Following heterologous virus challenge, the mice vaccinated with PGA/alum adjuvanted chimeric protein (1 μg) showed similar protection compared with those immunized with PGA/alum adjuvanted split vaccine. The pandemic vaccine is the most effective way to control the infection of pandemic influenza virus. However, it usually takes more than six months to develop influenza vaccine. If a highly effective heterosubtypic cross-reactive influenza vaccine is available, we could develop it for emergency use during an influenza pandemic. Thus, PGA/alum-adjuvanted chimeric vaccine may be a candidate universal vaccine for emergency use during influenza pandemics.

In summary, the chimeric protein is an influenza vaccine antigen that induces humoral and cellular immunity such as CTL and ADCC response. The cross-reactive effects of the chimeric protein were enhanced by PGA/alum. Thus, chimeric protein with PGA/alum cross-protected mice against heterologous influenza A viruses. Therefore, the PGA/alum-adjuvanted chimeric protein may be a promising universal influenza vaccine candidate.

## Supplemental Materials



Supplementary data for this paper are available on-line only at http://jmb.or.kr.
