# Supplementary material for: Influenza Chimeric Protein (3M2e-3HA2-NP) Adjuvanted with PGA/Alum Confers Cross-Protection against Heterologous Influenza A Viruses
Source: J Microbiol Biotechnol. 2020 Dec 2;31(2):304–16. doi: 10.4014/jmb.2011.11029 (PMC9705887; doi:10.4014/jmb.2011.11029)
Supplement: Supplementary file 1 [file jmb-31-2-304-supple.pdf]

J. Microbiol. Biotechnol.
https://doi.org/10.4014/jmb.2011.11029

Influenza Chimeric protein (3M2e-3HA2-NP) adjuvanted with PGA/alum confers cross-protection against heterologous influenza A viruses

Supplementary Figure Legends

FIGURE S1. Construction and confirmation of recombinant proteins (3M2e, 3HA2, and NP). (A) 3M2e, (B) 3HA2 and (C) NP genes were cloned into the pET21a(+) plasmid. Expression of purified (A) 3M2e, (B) 3HA2, and (C) NP was confirmed by SDS-PAGE.

FIGURE S2. Chimeric protein induces protective antibodies against PR8 virus. (A) Sera were collected from chimeric protein-vaccinated mice and heat-inactivated at 56 ºC for 1 h. Naïve BALB/c mice (n = 5) intranasally infected with 2 LD_{50} of PR8 virus mixed with immune sera. Body weight and survival rate were monitored for two weeks after PR8 virus infection. (B) HI titers of immune sera were measured against PR8 virus. Data are expressed as the mean ± SEM. Positive sera were obtained from mice infected with a sublethal dose (0.1 LD_{50}) two times at 2-week intervals.

FIGURE S3. PGA/alum-adjuvanted chimeric protein confers higher protection than γ-PGA-adjuvanted chimeric protein against PR8 virus. Female C57BL/6 mice (n = 5 per group) were i.m. immunized with (A) 1.2, (B) 1.8, and (C) 2.5 μg of chimeric protein with γ-PGA or PGA/alum three times at 2-week intervals. Fourteen days after the final vaccination, the mice were infected with 10 LD_{50} of PR8 virus. Body weight and survival rate were observed for

two weeks. C, chimeric protein.

FIGURE S4. PGA/alum increases cross-reactive antibodies of the chimeric protein against influenza A viruses. (A) BALB/c mice (n = 5) intranasally infected with mixtures of 2 LD_{50} (PR8, H3N2) or 3 LD_{50} (CA04) of influenza A viruses with immune sera. Body weight and survival rate were measured for two weeks after virus infection. (B) HI titers of immune sera against each influenza virus (PR8, CA04, or H3N2) were determined. Data are shown as the mean ± SEM. Positive sera were obtained from mice infected with a sublethal dose (0.1 LD_{50}) two times at 2-week intervals.

FIGURE S5. Activation of NK cells is involved in the protective effect of the PGA/alum-adjuvanted chimeric protein. C57BL/6 mice (n = 5) intramuscularly vaccinated with phosphate-buffered saline (PBS), chimeric, chimeric with alum, and chimeric with PGA/alum three times at 2-week intervals. Two weeks after the final immunization, sera were collected from immunized mice and heat-inactivated at 56 ºC for 1 h. (A) MDCK cells were infected with indicated influenza A viruses (MOI = 1), followed by incubation with immune sera. After 1 h, cells were harvested and stained with Alexa Fluor 488-conjugated goat anti-mouse IgG. Binding affinity of antibodies was detected by flow cytometry. (B) Recombinant protein 3HA2-coated plates were incubated with immune sera, followed by incubation with naïve NK cells with PE-conjugated anti-CD107a antibody in presence of monensin and brefeldin A at 37 ºC for 5 h. Cells were fixed and permeabilized with the BD Cytofix/Cytoperm™ kit, and stained with APC-conjugated anti-IFN-γ antibody. Stained cells were detected by flow

cytometry for expression of CD107a and IFN-γ. Data are representative of three independent experiments, and statistically significant differences were identified using one-way ANOVA/Bonferroni; *$p < 0.05$, **$p < 0.01$.

FIGURE S6. $CD8^+$ T cells are activated by the PGA/alum-adjuvanted chimeric protein. (A–C) C57BL/6 mice (n = 3) intramuscularly immunized with PBS, chimeric, chimeric with alum, or chimeric with PGA/alum three times at 2-week intervals. Two weeks after the last immunization, splenocytes were isolated from immunized mice and stimulated with 2000 $TCID_{50}$ of (A) UV-inactivated PR8 virus, (B) CA04 virus, or (C) H3N2 virus. Influenza virus-specific IFN-γ^+ $CD8^+$ T cells were detected by flow cytometry. Data are expressed as the mean ± SEM, and statistical significance was measured using one-way ANOVA/Bonferroni; *$p < 0.05$, **$p < 0.01$.

Figure S1

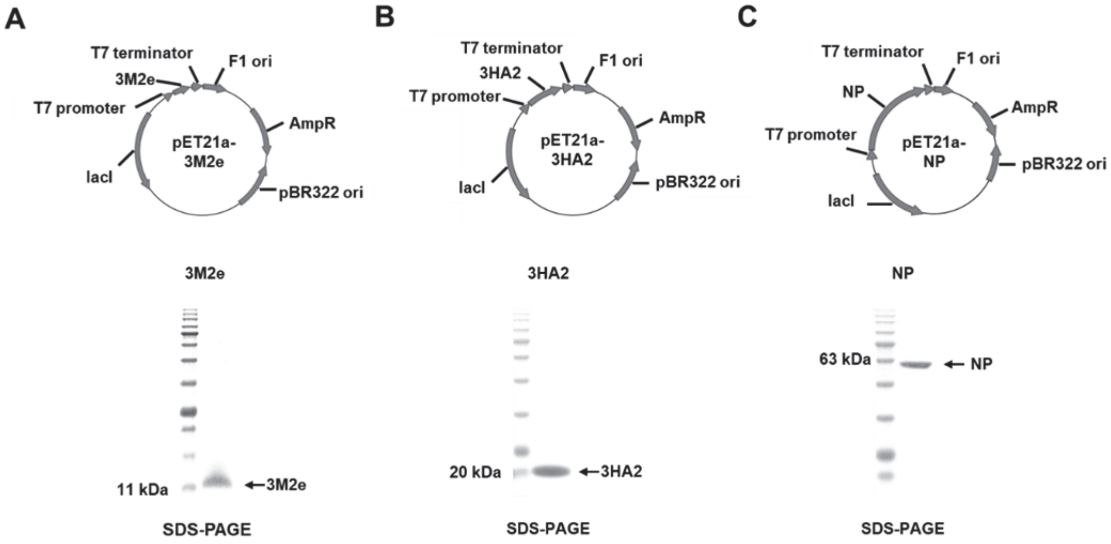
A
T7 terminator
3M2e
F1 ori
T7 promoter
AmpR
pET21a-
3M2e
lacI
pBR322 ori
3M2e
11 kDa
3M2e
SDS-PAGE
B
T7 terminator
3HA2
F1 ori
T7 promoter
AmpR
pET21a-
3HA2
lacI
pBR322 ori
3HA2
20 kDa
3HA2
SDS-PAGE
C
T7 terminator
F1 ori
NP
AmpR
T7 promoter
pET21a-
NP
pBR322 ori
lacI
NP
63 kDa
NP
SDS-PAGE

Figure S2

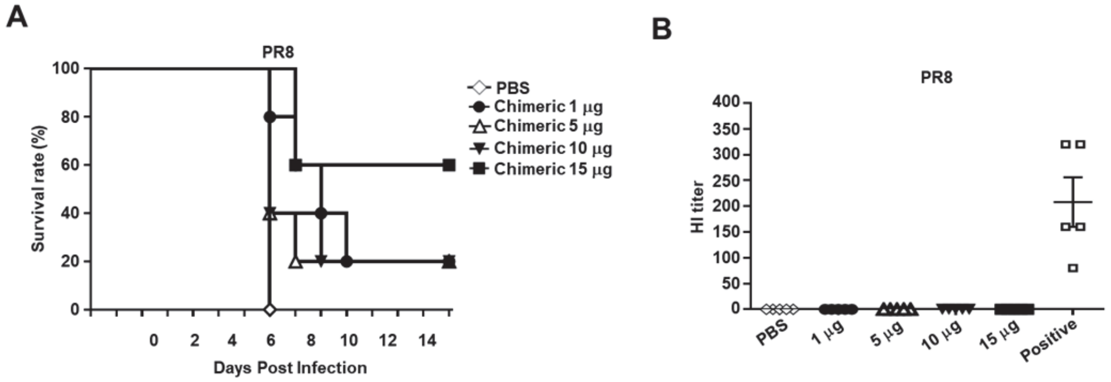
A
PR8
100
80
60
40
20
0
Survival rate (%)
0 2 4 6 8 10 12 14
Days Post Infection
PBS
Chimeric 1 μg
Chimeric 5 μg
Chimeric 10 μg
Chimeric 15 μg
B
PR8
400
350
300
250
200
150
100
50
0
HI titer
PBS
1 μg
5 μg
10 μg
15 μg
Positive

Figure S3

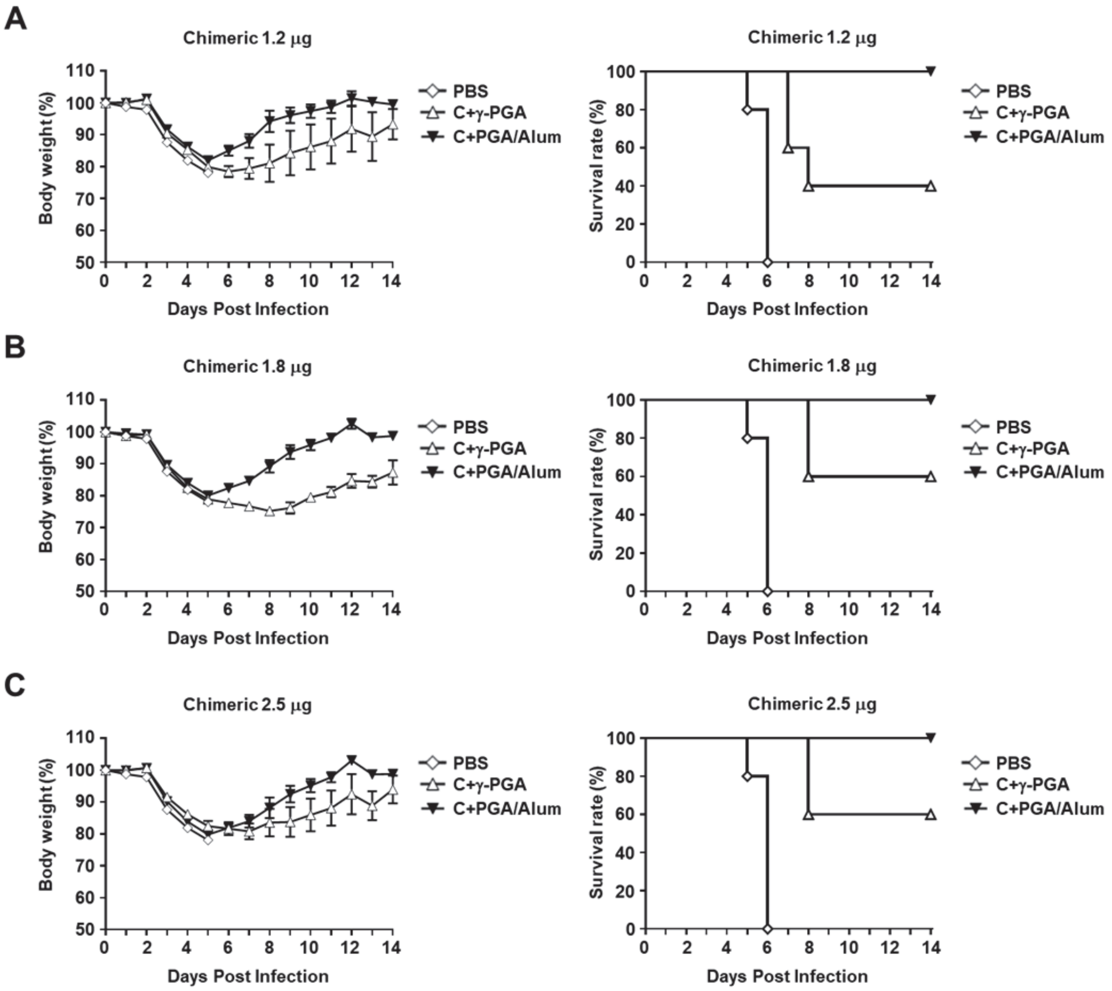
A
Chimeric 1.2 μg
Body weight (%)
Days Post Infection
Survival rate (%)
PBS
C+γ-PGA
C+PGA/Alum
B
Chimeric 1.8 μg
C
Chimeric 2.5 μg

Figure S4

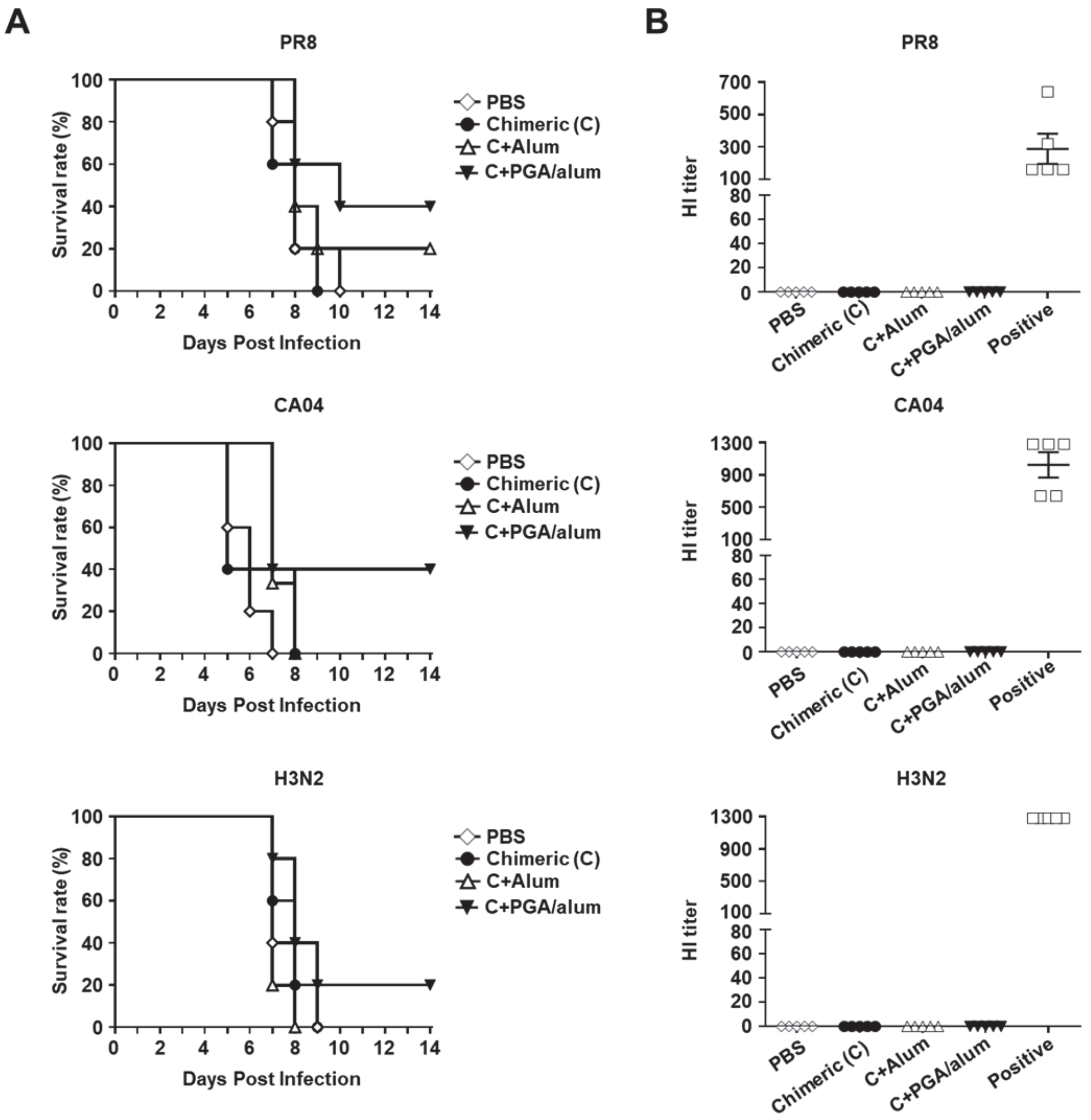

A
B
PR8
CA04
H3N2
Survival rate (%)
Days Post Infection
PBS
Chimeric (C)
C+Alum
C+PGA/alum
HI titer
Positive

Figure S5

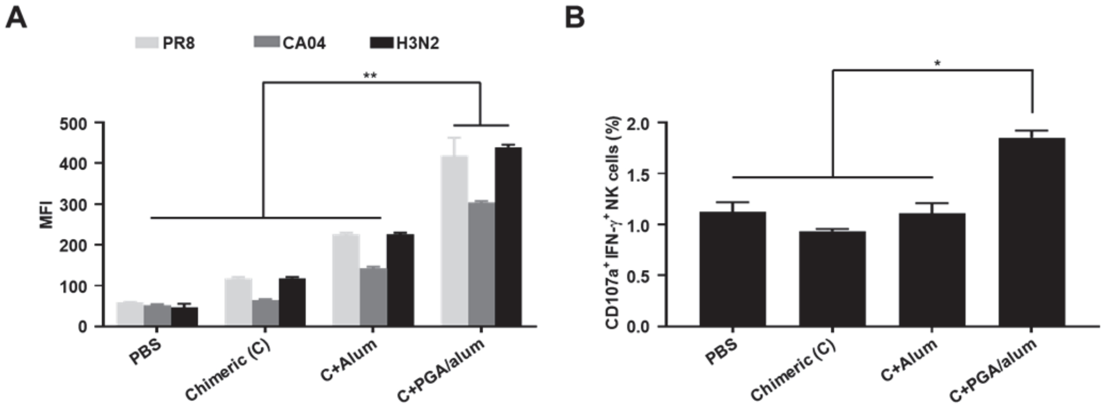
A
PR8
CA04
H3N2
**
MFI
500
400
300
200
100
0
PBS
Chimeric (C)
C+Alum
C+PGA/alum
B
*
CD107a+ IFN-γ+ NK cells (%)
2.0
1.5
1.0
0.5
0.0
PBS
Chimeric (C)
C+Alum
C+PGA/alum

Figure S6

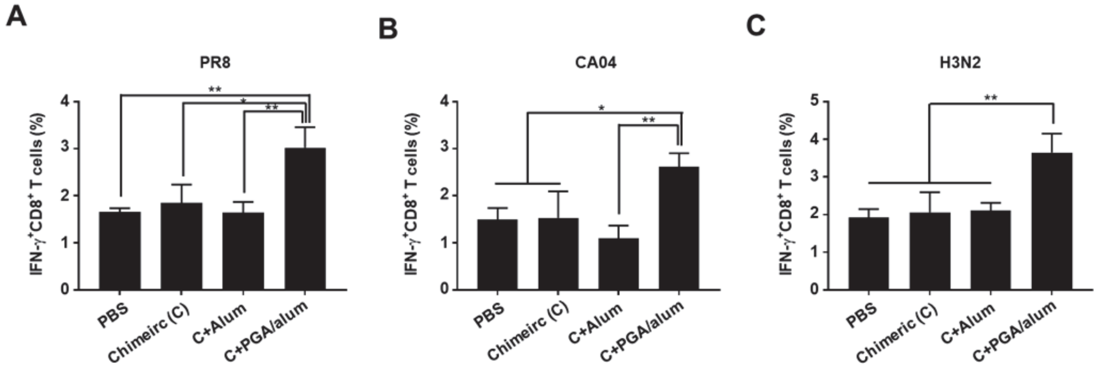
A
PR8
IFN-γ⁺CD8⁺ T cells (%)
PBS
Chimeirc (C)
C+Alum
C+PGA/alum
B
CA04
IFN-γ⁺CD8⁺ T cells (%)
PBS
Chimeirc (C)
C+Alum
C+PGA/alum
C
H3N2
IFN-γ⁺CD8⁺ T cells (%)
PBS
Chimeric (C)
C+Alum
C+PGA/alum
